# Naloxone and Patient Outcomes in Out-of-Hospital Cardiac Arrests in California

**DOI:** 10.1001/jamanetworkopen.2024.29154

**Published:** 2024-08-20

**Authors:** David G. Dillon, Juan Carlos C. Montoy, Daniel K. Nishijima, Sara Niederberger, James J. Menegazzi, Jeremy Lacocque, Robert M. Rodriguez, Ralph C. Wang

**Affiliations:** 1 Department of Emergency Medicine, University of California, Davis; 2 Department of Emergency Medicine, University of California, San Francisco; 3 Department of Emergency Medicine, University of Pittsburgh, Pennsylvania

## Abstract

**Importance:**

The incidence of opioid-associated out-of-hospital cardiac arrest (OA-OHCA) has grown from less than 1% of OHCA in 2000 to between 7% and 14% of OHCA in recent years; American Heart Association (AHA) protocols suggest that emergency medical service (EMS) clinicians consider naloxone in OA-OHCA. However, it is unknown whether naloxone improves survival in these patients or in patients with undifferentiated OHCA.

**Objective:**

To evaluate the association of naloxone with clinical outcomes in patients with undifferentiated OHCA.

**Design, Setting, and Participants:**

Retrospective cohort study of EMS-treated patients aged 18 or older who received EMS treatment for nontraumatic OHCA in 3 Northern California counties between 2015 and 2023. Data were analyzed using propensity score-based models from February to April 2024.

**Exposure:**

EMS administration of naloxone.

**Main Outcomes and Measures:**

The primary outcome was survival to hospital discharge; the secondary outcome was sustained return of spontaneous circulation (ROSC). Covariates included patient and cardiac arrest characteristics (eg, age, sex, nonshockable rhythm, any comorbidity, unwitnessed arrest, and EMS agency) and EMS clinician determination of OHCA cause as presumed drug-related.

**Results:**

Among 8195 patients (median [IQR] age, 65 [51-78] years; 5540 male [67.6%]; 1304 Asian, Native Hawaiian, or Pacific Islander [15.9%]; 1119 Black [13.7%]; 2538 White [31.0%]) with OHCA treated by 5 EMS agencies from 2015 to 2023, 715 (8.7%) were believed by treating clinicians to have drug-related OHCA. Naloxone was administered to 1165 patients (14.2%) and was associated with increased ROSC using both nearest neighbor propensity matching (absolute risk difference [ARD], 15.2%; 95% CI, 9.9%-20.6%) and inverse propensity–weighted regression adjustment (ARD, 11.8%; 95% CI, 7.3%-16.4%). Naloxone was also associated with increased survival to hospital discharge using both nearest neighbor propensity matching (ARD, 6.2%; 95% CI, 2.3%-10.0%) and inverse propensity–weighted regression adjustment (ARD, 3.9%; 95% CI, 1.1%-6.7%). The number needed to treat with naloxone was 9 for ROSC and 26 for survival to hospital discharge. In a regression model that assessed effect modification between naloxone and presumed drug-related OHCA, naloxone was associated with improved survival to hospital discharge in both the presumed drug-related OHCA (odds ratio [OR], 2.48; 95% CI, 1.34-4.58) and non–drug-related OHCA groups (OR, 1.35; 95% CI, 1.04-1.77).

**Conclusions and Relevance:**

In this retrospective cohort study, naloxone administration as part of EMS management of OHCA was associated with increased rates of ROSC and increased survival to hospital discharge when evaluated using propensity score–based models. Given the lack of clinical practice data on the efficacy of naloxone in OA-OHCA and OHCA in general, these findings support further evaluation of naloxone as part of cardiac arrest care.

## Key Points

**Question:**

Is naloxone administration during out-of-hospital cardiac arrest (OHCA) associated with return of spontaneous circulation or survival to hospital discharge?

**Findings:**

In this cohort study of 8195 patients with OHCA treated in 3 Northern California counties between 2015 and 2023, emergency medical service administration of naloxone was associated with significantly improved outcomes. The number needed to treat with naloxone was 9 for return of spontaneous circulation and 26 for survival to hospital discharge.

**Meaning:**

These findings support further evaluation of naloxone as part of cardiac arrest care.

## Teaser

This cohort study evaluates the association of naloxone administration with clinical outcomes in patients with out-of-hospital cardiac arrests in California.

## Introduction

Out-of-hospital cardiac arrest (OHCA) is a growing public health problem with a poor prognosis. Treatment guidelines for OHCA exist, but evidence-based therapies are limited, and nearly 90% of the 356 000 cases of OHCA in the US each year are fatal.^1^ Over the past 2 decades, an increasing proportion of OHCA has been shown to be secondary to drug overdose,^2^ and the incidence of opioid-associated (OA) OHCA grew from less than 1% of all OHCA in 2000 to between 7% and 14% of OHCA by the end of the 2010s.^3,4,5,6^ The prevalence of OA-OHCA has continued to rise during the COVID-19 pandemic,^7^ and we have found that 17.4% of emergency medical services (EMS)–attended OHCA in San Francisco County in 2023 were suspected to be drug-related.^8^

The American Heart Association (AHA) defines OA-OHCA as OHCA precipitated by the use of opioids, with or without cointoxicants.^2^ In OA-OHCA, hypopnea leads to hypoxia, which in turn causes a progressive reduction in cardiac output, hypotension, bradycardia, and finally, cardiac arrest. This gradual progression to cardiac arrest in OA-OHCA—several minutes as compared with shorter intervals with sudden events, such as arrythmia-induced cardiac arrest—may offer greater opportunity for targeted rescue interventions, like assisted ventilation and the opioid receptor competitive antagonist (ie, reversal agent) naloxone.^2^

While naloxone is known to be beneficial in drug overdose without concurrent cardiac arrest, it is unknown whether naloxone is beneficial to patients with OA-OHCA.^2,9^ Naloxone reverses opioid-associated apnea and altered level of consciousness, and also has effects on blood pressure and cardiac rhythm that confer biological plausibility for use in OA-OHCA.^9,10,11^ The current leading hypothesis is that naloxone reverses opioid-related myocardial depression and stimulates catecholamine release, with consequent augmentation of heart rate and blood pressure.^2,11^

Current AHA guidelines for the treatment of OHCA recommend that EMS clinicians treating patients with known or suspected OA-OHCA should consider naloxone, but do not specifically recommend naloxone administration in these cases. Furthermore, most EMS protocols are not written to differentiate OA-OHCA from other causes of OHCA.^12^ The AHA recently identified the rigorous evaluation of naloxone’s efficacy in OA-OHCA as an important knowledge gap; however, no prospective studies that we know of have been conducted to assess the efficacy of naloxone in OA-OHCA or undifferentiated OHCA in general.^2^ We sought to evaluate the association between naloxone administration and clinical outcomes for patients with OHCA. Specifically, we sought to determine whether naloxone was associated with improved rates of return of spontaneous circulation (ROSC) and survival to hospital discharge.

## Methods

### Study Participants, Setting, and Data Collection

We conducted a retrospective cohort study of patients with OHCA who were treated by EMS clinicians in Sacramento County, San Francisco County, or Yolo County, California between 2015 and 2023. We obtained data from the Sacramento City Fire Department, San Francisco County EMS Agency, and Yolo County EMS Agency collected for the Cardiac Arrest Registry to Enhance Survival, a prospective registry of OHCA established by the US Centers for Disease Control and Prevention and Emory University.^13^ The San Francisco County EMS Agency and Yolo County EMS Agency are regulatory agencies that have medical oversight of the EMS agencies within their jurisdictions; Sacramento Fire is an EMS agency serving the City of Sacramento. Standardized international Utstein definitions for reporting clinical variables and outcomes associated with cardiac arrest were used to ensure data uniformity.^14^ Participant race and ethnicity were reported to describe the overall patient population. This work was approved by the University of California, San Francisco institutional review board, and we adhered to Strengthening the Reporting of Observational Studies in Epidemiology (STROBE) reporting guidelines. Informed consent was waived because the data were deidentified.

Participants included adults with nontraumatic OHCA in whom resuscitation was attempted by EMS clinicians. We excluded patients (1) less than 18 years old, or (2) who were missing data regarding the administration of medications. Our primary outcome of interest was survival to hospital discharge, defined as being discharged from the hospital as recorded in the electronic health record.^14^ Our secondary outcome of interest was sustained return of spontaneous circulation (ROSC), defined as having a detectable pulse for at least 20 minutes or at the end of EMS care. Our exposure was EMS-administered naloxone during treatment of OHCA, identified using the medication administration record. The exposed group received naloxone and the control group did not. We included covariates associated with the exposure and outcomes based on our review of the literature: age, sex, initial cardiac rhythm, comorbid conditions, whether the OHCA was witnessed, and whether the cause of arrest was drug-related.^2,15,16^ Additional standard covariates were considered, including bystander CPR, location of arrest, and use of an automatic external defibrillator, however they were ultimately excluded from the analyses due to collinearity. A cardiac arrest was identified as drug-related when the arrest was caused by a known or presumed overdose of legal or illegal substances. The determination of whether OHCA was drug-related was made by the treating EMS clinicians on a case-by-case basis. We also included EMS agency as a covariate to account for regional and agency-level differences in naloxone use and OHCA survival. All covariates were abstracted directly from the EMS and hospital electronic health records.

### Statistical Analysis

We present binary data as counts and percentages, continuous data as means and SDs or medians and IQRs, and between-group differences as absolute risk differences (ARDs), risk ratios (RRs), or odds ratios (ORs) with 95% CIs. Data were analyzed using logistic regression models that included variables for age, sex, drug-related OHCA cause, nonshockable rhythm (asystole, pulseless electrical activity, or other nonshockable rhythm), any comorbidity, unwitnessed arrest, and EMS agency. We used mixed-effects models to account for the hierarchical structure of the data, with clustering at the level of the EMS agency. Robust SEs were used where appropriate.

To reduce the risk of selection bias, we generated 2 propensity score models: inverse probability-weighted regression adjustment, and nearest neighbor propensity score matching. For the inverse probability-weighted regression adjustment model, we performed a propensity score regression using the variables listed previously. Estimated parameters of the exposure model were used to compute inverse-probability weights, which were then used to fit weighted regression models of the exposure-specific estimated outcome for each patient. Exposure-specific estimated outcomes were compared to estimate the average exposure effect. For the nearest neighbor propensity score matching model, we generated propensity scores from a regression model using the same variables as the inverse probability-weighted adjustment model. We then matched patients in a 1:1 fashion using a caliper matching method without replacement and a caliper width of 0.1 SD of the logit of the propensity score. The exposed and not exposed groups were comparable for included variables for both propensity score models (−0.1 < standardized mean difference < 0.1). The eFigure in Supplement 1 shows adequate propensity score overlap.

We also performed 2 sets of additional analyses. The first examined the average treatment effect on the treated (ATET). ATET estimates the effect of an intervention (ie, naloxone) by restricting the sample to those patients who received it and estimating the difference in outcome conferred by the treatment compared with the counterfactual of not having had the treatment. The second analysis was a mixed-effects regression model using the covariates listed previously that also included an interaction term for naloxone administration and presumed drug-related OHCA to account for potential effect modification between these 2 variables. This allowed us to assess whether an interaction was present, and to estimate the association between naloxone and clinical outcomes in 2 groups: the presumed drug-related OHCA subgroup and the non–drug related OHCA subgroup. All hypothesis tests were 2-sided and used an a priori level of significance of .05. Data were analyzed using Stata software version 17 (StataCorp). Data were analyzed from February to April 2024.

## Results

### Study Participants

We identified 8339 medical records of patients treated for nontraumatic cardiac arrest between 2015 and 2023. After medical record review, 122 medical records were excluded for not meeting our predefined age inclusion criteria (eg, age ≥18 years) and 22 medical records were excluded for having incomplete exposure data (0.3% of the total number of medical records evaluated). Of 8195 patients included in our analyses, most were male (5540 patients [ 67.6%]), and their median (IQR) age was 65 (51-78) years (Table 1). There were a total of 1304 Asian, Native Hawaiian, or Pacific Islander patients (15.9%); 1119 Black patients (13.7%); and 2538 White patients (31.0%). We found 6707 patients (81.8%) had nonshockable cardiac rhythms, such as asystole or pulseless electrical activity, and 1488 (18.2%) had shockable rhythms. Seven hundred and fifteen participants (8.7%) were identified as presumed drug-related OHCA by the treating EMS clinician.

**Table 1.** Baseline Patient and Cardiac Arrest Characteristics, Stratified by Naloxone Administration

Characteristic	Patients, No. (%)			***P* value** ^a^
	Overall (N = 8195)	Naloxone (n = 1165)	No naloxone (n = 7030)	
Patient characteristics				
Age, median (IQR), y	65 (51-78)	46.5 (35-60)	67 (55-80)	<.001
Sex				
Male	5540 (67.6)	880 (75.5)	4660 (66.3)	<.001
Female	2652 (32.4)	283 (24.3)	2369 (33.7)	
Nonbinary	1 (<0.1)	1 (0.1)	0	
Unknown	2 (<0.1)	1 (0.1)	1 (<0.1)	
Race and ethnicity				
American Indian/Alaska Native	35 (0.4)	6 (0.5)	29 (0.4)	<.001
Asian/Native Hawaiian/Pacific Islander	1304 (15.9)	64 (5.5)	1240 (17.6)	
Black	1119 (13.7)	249 (21.4)	870 (12.4)	
Hispanic/Latino	663 (8.1)	152 (13.1)	511 (7.3)	
White	2538 (31.0)	371 (31.9)	2167 (30.8)	
More than 1 race	19 (0.2)	7 (0.6)	12 (0.2)	
Unknown	2517 (30.7)	316 (27.1)	2201 (31.3)	
Comorbidities				
Cancer	328 (4.0)	21 (1.8)	307 (4.4)	<.001
Diabetes	1323 (16.1)	82 (7.0)	1241 (17.7)	
Heart disease	1221 (14.9)	70 (6.0)	1151 (16.4)	
Hypertension	1794 (21.9)	101 (8.7)	1693 (24.1)	
Hyperlipidemia	453 (5.5)	23 (2.0)	430 (6.1)	
Kidney disease	238 (2.9)	11 (0.9)	227 (3.2)	
Respiratory disease	505 (6.2)	47 (4.0)	458 (6.5)	
Stroke	358 (4.4)	14 (1.2)	344 (4.8)	
Any comorbidity	3588 (43.8)	237 (20.3)	3351 (47.7)	
Cardiac arrest characteristics				
EMS agency				<.001
San Francisco Fire Department	4028 (49.2)	547 (47.0)	3481 (49.5)	
King American, San Francisco	643 (7.9)	153 (13.1)	490 (7.0)	
Global Medical Response, San Francisco	560 (6.8)	118 (10.1)	442 (6.3)	
Sacramento City Fire Department	2061 (25.2)	256 (22.0)	1805 (25.7)	
Global Medical Response, Yolo	903 (11.0)	91 (7.8)	812 (11.6)	
Location of arrest				
Home/living facility	6037 (73.7)	645 (55.4)	5392 (76.7)	<.001
Street	1201 (14.7)	365 (31.3)	836 (11.9)	
Public/commercial building	595 (7.3)	126 (10.8)	469 (6.7)	
Other	362 (4.4)	29 (2.5)	333 (4.7)	
Witnessed arrest				
Witnessed	4481 (54.7)	534 (45.8)	3947 (56.1)	<.001
Unwitnessed	3714 (45.3)	631 (54.2)	3083 (43.9)	
Initial rhythm				
Asystole	4310 (52.6)	655 (56.2)	3655 (52.0)	<.001
PEA	2186 (26.7)	334 (28.7)	1852 (26.4)	
Other unshockable rhythm	211 (2.6)	41 (3.5)	170 (2.4)	
Ventricular tachycardia	142 (1.7)	17 (1.5)	125 (1.8)	
Ventricular fibrillation	1235 (15.1)	107 (9.2)	1128 (16.0)	
Other shockable rhythm	111 (1.4)	11 (0.9)	100 (1.4)	
Presumed cause of arrest				
Drug-related	715 (8.7)	471 (40.4)	244 (3.5)	<.001
Not drug-related	7480 (91.3)	694 (59.6)	6786 (96.5)	

Abbreviations: EMS, emergency medical service; PEA, pulseless electrical activity.

^a^ *P* values describe differences between the naloxone exposed and unexposed groups. *P* ≤ .05 indicates statistical significance.

### Naloxone Administration by EMS Clinicians

Naloxone was administered in 1165 of OHCA cases (14.2%) (Table 1). The demographic and arrest characteristics of the patients treated with naloxone differed substantially from the nonexposed group; the naloxone group was younger, more likely to be male, and had fewer comorbidities compared with patients who did not receive naloxone. Naloxone was also preferentially given in presumed drug-related OHCA cases compared with all other OHCA, although 694 OHCA cases that received naloxone (59.6%) were not thought to be drug-related. Naloxone administration was more common in patients with nonshockable rhythms and in unwitnessed arrest. The naloxone group was more likely to obtain ROSC, survive to hospital admission, and survive to hospital discharge (Table 2). In the naloxone group, 402 of 1165 patients (34.5%) obtained ROSC, while in the nonexposed group, 1609 of 7030 (22.9%) obtained ROSC. In the naloxone group, 185 of 1165 patients (15.9%) survived to hospital discharge, while in the nonexposed group, 682 of 7030 (9.7%) survived to hospital discharge.

**Table 2.** Interventions Administered and Cardiac Arrest Outcome, Stratified by Naloxone Administration

Intervention and Outcome	Patients, No. (%)			*P* value^a^
	Overall (N = 8195)	Naloxone (n = 1165)	No naloxone (n = 7030)	
Interventions administered				
Epinephrine administered				
Yes	7124 (86.9)	1048 (90.0)	6076 (86.4)	.001
No	1071 (13.1)	117 (10.0)	954 (13.6)	
Who performed initial CPR				
Family member	1187 (14.5)	121 (10.4)	1066 (15.2)	<.001
Bystander	1491 (18.2)	188 (16.1)	1303 (18.5)	
EMS/first responder	5516 (67.3)	856 (73.5)	4660 (66.3)	
Missing	1 (<0.1)	0	1 (<0.1)	
Airway placed in field				
Yes	1969 (24.0)	212 (18.2)	1757 (25.0)	<.001
No	5395 (65.8)	821 (70.5)	4574 (65.1)	
Missing	831 (10.1)	132 (11.4)	699 (9.9)	
Cardiac arrest outcome				
Resuscitation terminated in field				
Yes	2987 (36.5)	285 (24.7)	2702 (38.4)	<.001
No	5208 (63.6)	880 (75.5)	4328 (61.6)	
Admitted to hospital from ED				
Yes	2714 (33.1)	533 (45.8)	2181 (31.2)	<.001
No	5357 (65.4)	591 (50.7)	4766 (67.8)	
Missing	124 (1.5)	41 (3.5)	83 (1.2)	
Sustained ROSC^b^				
Yes	2011 (24.5)	402 (34.5)	1609 (22.9)	<.001
No	6183 (75.5)	762 (65.4)	5421 (77.1)	
Missing	1 (<0.1)	1 (0.1)	1 (<0.1)	
Discharged from hospital				
Yes	867 (10.6)	185 (15.9)	682 (9.7)	<.001
No	7202 (87.9)	938 (80.5)	6264 (89.1)	
Missing	126 (1.5)	42 (3.6)	84 (1.2)	

Abbreviations: CPR, cardiopulmonary resuscitation; ED, emergency department; EMS, emergency medical service; ROSC, return of spontaneous circulation.

^a^ *P* values describe differences between the naloxone exposed and unexposed groups. *P* ≤ .05 indicates statistical significance.

^b^ Sustained ROSC defined as having a detectable pulse for at least 20 minutes or at the end of EMS care.

Patients with presumed drug-related OHCA (OR, 8.40; 95% CI, 6.93-10.10) and patients with nonshockable cardiac rhythms (OR, 1.59; 95% CI, 1.30-1.96) had higher odds of receiving naloxone in our cohort in the adjusted regression model (Table 3). Conversely, older patient age and the presence of comorbidities (OR, 0.63; 95% CI, 0.53-0.76) were associated with lower odds of receiving naloxone.

**Table 3.** Odds of Receiving Naloxone by Patient and Cardiac Arrest Characteristics^a^

Characteristic	OR (95% CI)	aOR (95% CI)
Presumed drug-related OHCA	19.1 (16.1-22.8)	8.40 (6.93-10.10)
Age, y		
18-50	1 [Reference]	1 [Reference]
51-64	0.32 (0.28-0.38)	0.50 (0.42-0.59)
65-78	0.13 (0.11-0.16)	0.26 (0.21-0.33)
≥79	0.04 (0.03-0.06)	0.10 (0.07-0.13)
Male sex	1.56 (1.35-1.80)	1.21 (1.02-1.42)
Nonshockable rhythm	1.86 (1.53-2.25)	1.59 (1.30-1.96)
Any comorbidity	0.26 (0.23-0.31)	0.63 (0.53-0.76)
Unwitnessed OHCA	1.52 (1.35-1.73)	1.06 (0.91-1.22)

Abbreviations: aOR, adjusted odds ratio; OHCA, out-of-hospital cardiac arrest; OR, odds ratio.

^a^ ORs greater than 1 represent an increased odds of receiving naloxone in our cohort. A mixed-effects regression model was used to calculate aORs and included variables for age (quartiles based on cohort median and interquartile range), presumed drug-related OHCA cause, sex, nonshockable rhythm, any comorbidity, and unwitnessed OHCA. Emergency medical service agency was used as the group level variable.

### Naloxone Association With ROSC and Survival

To account for imbalances in the exposed and nonexposed groups, we employed both propensity score-based nearest neighbor matching and inverse probability-weighted regression analysis techniques. We first estimated the unadjusted absolute risk difference (ARD) for the association of naloxone with ROSC (ARD, 11.6%; 95% CI, 8.7%-14.6%) and survival to hospital discharge (ARD, 6.7%; 95% CI, 4.4%-8.9%) (Table 4). Naloxone was associated with ROSC using both nearest neighbor propensity matching (ARD, 15.2%; 95% CI, 9.9%-20.6%) and inverse propensity–weighted regression adjustment (ARD, 11.8%; 95% CI, 7.3%-16.4%). Naloxone was also associated with increased survival to hospital discharge using both nearest neighbor propensity matching (ARD, 6.2%; 95% CI, 2.3%-10.0%) and inverse propensity–weighted regression adjustment (ARD, 3.9%; 95% CI, 1.1%-6.7%). Risk ratios for these associations showed similarly positive estimates across all models (Table 4). When restricted to assessing the average association of naloxone administration with clinical outcomes in treated individuals, the risk ratios from our ATET models have higher point estimates compared with the analyses of the whole cohort.

**Table 4.** Propensity Score–Adjusted Models of Return of Spontaneous Circulation and Survival to Hospital Discharge for Patients Receiving Naloxone During Emergency Medical Service–Attended Out-of-Hospital Cardiac Arrest

	Return of spontaneous circulation		Survival to hospital discharge	
	Absolute risk difference, % (95% CI)	RR (95% CI)^a^	Absolute risk difference, % (95% CI)	RR (95% CI)^a^
Unadjusted effect size estimate	11.6 (8.7-14.6)	1.51 (1.38-1.65)	6.7 (4.4-8.9)	1.68 (1.44-1.95)
Nearest neighbor propensity matching	15.2 (9.9-20.6)	1.67 (1.43-1.90)	6.2 (2.3-10.0)	1.63 (1.24-2.02)
Inverse probability-weighted regression adjustment	11.8 (7.3-16.4)	1.55 (1.36-1.77)	3.9 (1.1-6.7)	1.51 (1.22-1.87)
Treatment effect in the treated				
Nearest neighbor propensity matching	15.4 (11.3-19.4)	1.80 (1.58-2.02)	8.2 (5.2-11.3)	2.01 (1.58-2.43)
Inverse probability-weighted regression adjustment	15.5 (11.7-19.2)	1.80 (1.73-1.88)	7.8 (5.1-10.4)	1.89 (1.77-2.01)

Abbreviation: RR, risk ratio.

^a^ RRs are presented for patients receiving naloxone compared with those who do not receive naloxone. RRs greater than 1 represent a protective association for naloxone and risk ratios less than 1 represent a harmful association.

In our analysis using a mixed-effects regression model that included an interaction term between naloxone administration and presumed drug-related OHCA, naloxone administration was associated with improved clinical outcomes in both the presumed drug-related OHCA and non–drug related OHCA groups, and there was a statistically significant interaction between naloxone and drug-related causes (ROSC χ^2^_3_ *=* 126.2; *P* < .001; survival χ^2^_3_ *=* 64.0; *P* < .001) (Table 5). Compared with the subgroup of nonexposed non–drug related OHCAs, naloxone was associated with improved rates of ROSC in both non–drug related OHCAs (OR, 1.61; 95% CI, 1.34-1.94) and drug-related OHCAs (OR, 2.45; 95% CI, 1.56-3.83). Naloxone was also associated with increased survival to hospital discharge in drug-related OHCAs (OR, 2.48; 95% CI, 1.34-4.58) and associated with increased survival to hospital discharge in non–drug related OHCAs (OR, 1.35; 95% CI, 1.04-1.77).

**Table 5.** Adjusted Logistic Regression Model of Return of Spontaneous Circulation and Survival to Hospital Discharge for Patients Receiving Naloxone During Emergency Medical Service (EMS)–Attended Out-of-Hospital Cardiac Arrest (OHCA)^a^

Characteristic	OR (95% CI)	
	Return of spontaneous circulation	Survival to hospital discharge
Non–drug related OHCA not treated with naloxone^b^	1 [Reference]	1 [Reference]
Presumed drug-related OHCA not treated with naloxone^c^	0.81 (0.55-1.18)	0.91 (0.54-1.53)
Non–drug related OHCA treated with naloxone^d^	1.61 (1.34-1.94)	1.35 (1.04-1.77)
Presumed drug-related OHCA treated with naloxone^e^	2.45 (1.56-3.83)	2.48 (1.34-4.58)
Age, y		
18-50	1 [Reference]	1 [Reference]
51-64	1.10 (0.94-1.29)	1.08 (0.89-1.32)
65-78	1.30 (1.11-1.54)	0.79 (0.63-0.99)
≥79	1.27 (1.07-1.51)	0.30 (0.23-0.41)
Sex (male)	0.81 (0.73-0.91)	0.88 (0.74-1.05)
Nonshockable rhythm	0.57 (0.50-0.65)	0.18 (0.15-0.21)
Unwitnessed arrest	0.47 (0.42-0.53)	0.36 (0.30-0.43)
Any comorbidity	0.85 (0.76-0.96)	0.73 (0.62-0.87)

Abbreviation: OR, odds ratio.

^a^ Odds ratios greater than 1 represent a protective association and odds ratios less than 1 represent a harmful association. Clustering of data was accounted for using a mixed-effects model with EMS agency as the group variable.

^b^ An interaction term for naloxone administration and presumed drug-related cause was included in the model. The group of patients where neither variable was present was used as a joint reference category.

^c^ The association of presumed drug-related cause without naloxone administration.

^d^ The association of naloxone administration without a presumed drug-related cause.

^e^ The joint association when both variables were present. There was a statistically significant interaction between naloxone administration and presumed drug-related cause for both return of spontaneous circulation (*P* < .001) and survival to hospital discharge (*P* < .001).

## Discussion

In this retrospective cohort study of adult patients with OHCA treated in 3 Northern California counties between 2015 and 2023, EMS administration of naloxone was associated with an 11.8–percentage point absolute increase in ROSC and a 3.9–percentage point absolute increase in patient survival to hospital discharge. These absolute risk differences translate to a number needed to treat (NNT) with naloxone of 9 for ROSC and 26 for survival to hospital discharge.

Our study represents one of the first large-scale evaluations of the association of naloxone with OHCA outcomes in clinical practice. Using propensity score-based models, we found that naloxone was associated with increased rates of ROSC and survival to hospital discharge in patients with undifferentiated OHCA. In additional analyses examining the average treatment effect on the treated group (eg, patients who tended to be younger, have unwitnessed arrests, and who were presumed by EMS to have drug-related OHCA),^17^ we found a larger positive association between naloxone and improved clinical outcomes compared with the main analyses. This suggests that the benefit of liberal naloxone therapy would likely be more pronounced in communities with a higher underlying risk of drug-related OHCA. Our results from the logistic regression models that included an interaction term between naloxone administration and suspected drug-related OHCA confirmed these findings. We detected a significant effect modification in the drug-related cause subgroup, although the associations between naloxone and improved clinical outcomes remained statistically significant in both drug-related and non–drug related OHCA subgroups.

While there are no published randomized trials of naloxone in OA-OHCA that we know of,^2^ there are multiple studies in animal models that suggest biologic plausibility for naloxone in improving outcomes in patients who experience overdose without a pulse.^18,19,20^ The specific underlying physiology for these results remains unclear, but the leading theory is that naloxone reverses opioid-induced myocardial depression while also triggering the release of catecholamines, thereby increasing heart rate, blood pressure, and respiratory drive.^2,11^ In humans, the use of naloxone in OHCA has been described in case reports and published abstracts, with preliminary analyses that suggest naloxone is associated with improved clinical outcomes.^21,22,23,24^ Our findings support these positive associations between naloxone, ROSC, and survival.

We also observed a weak association between naloxone and improved clinical outcomes in the non–drug related OHCA group. The mechanism of naloxone in this subgroup is unclear, but naloxone has been shown in a Cochrane review of 6 clinical trials to increase mean arterial blood pressure in shock by reversing endogenous opioids.^10,25,26,27^ These effects may confer improved outcomes in both OA-OHCA and all cause OHCA. Alternatively, the observed association may be explained by residual confounding or selection bias. For example, heterogeneity in defining drug-related OHCAs may have led to systematic misclassification of drug-related OHCA as non–drug related OHCA, as this variable was based solely on EMS clinician impression. Indeed, our data likely misclassify a nontrivial number of unrecognized drug-related OHCAs, as previously published results suggest that 1 in 6 presumed cardiac OHCA deaths were actually occult drug-related cardiac arrests.^28^

It is important to place our findings into the context of current clinical practice, which is notable for significant practice variation and the need for EMS clinicians to make quick decisions about resuscitation treatments with limited clinical information. Overall, 1 in 7 patients in our cohort received naloxone—two-thirds of patients with presumed drug-related OHCA and 1 in 10 patients with a non–drug related arrest cause. These results are similar to previously published rates, which show that naloxone was given in 13.5% of all medical OHCAs and 55% of presumed drug-related OHCAs that were included in the Cardiac Arrest Registry to Enhance Survival (CARES).^29^ These patterns may reflect ambiguity in the current AHA guidelines, which only recommend that naloxone be considered when treating a patient in OHCA.^30^ Indeed, fewer than 23% of EMS systems in the US mention naloxone in their cardiac arrest protocols, and 6% specifically state that naloxone should not be given.^12^ This ambiguity around naloxone administration in OHCA protocols likely reflects the lack of prospective evidence to define the role of naloxone in treating patients in cardiac arrest. Additional research is necessary to assess the effectiveness of naloxone in OA-OHCA and OHCA in general.

### Limitations

Our analysis has several limitations, many of which derive from the observational nature of our study, which may introduce potential for bias. Although we attempted to account for anticipated biases and confounding in our analysis plan, full adjustment for all confounding factors is not possible. Selection bias, in which EMS clinicians were more likely to administer naloxone to patients with suspected drug-related OHCA, could influence our results, as this population has been found to have better clinical outcomes compared with those with non–drug related OHCA.^29,31,32,33^ Similarly, younger patients with fewer comorbidities were more likely to receive naloxone and are independently more likely to survive to hospital discharge.^34^ In contrast, naloxone was more likely to be administered to patients with nonshockable rhythms, which are negatively associated with survival.^35^ There is also the potential for resuscitation time bias, in which refractory cases of cardiac arrest tend to have worse outcomes but are more likely to receive additional medications during a prolonged resucitation.^36^ We attempted to adjust for potential bias in our analysis through the use of propensity score-based models and analyses that included interaction terms and evaluated ATET, as these methods have been previously used successfully to compare exposed and nonexposed treatment groups with potentially differing underlying characteristics.^37,38^ While our findings were robust across all models, we were nevertheless unable to account for confounders that were not included in EMS reports. Similarly, we do not have data for why naloxone was given in any individual case, including why naloxone was given to a notable number of patients that were ultimately not presumed to have drug-related cardiac arrest. Thus, our results should be viewed in the appropriate context, and interventional studies are needed before ascribing causality to the identified associations between naloxone and improved clinical outcomes.

Our data were unable to differentiate routes of naloxone administration (eg, intravenous, intraosseous, or intranasal), which may influence the drug’s efficacy.^39^ Furthermore, we did not have data on the timing of the naloxone administration during OHCA resuscitation, which could introduce immortal person-time bias that we are unable to directly address. We were also unable to account for naloxone administered by bystanders or non-EMS first responders (eg, police or firefighters) during the resuscitation effort. Our assumption in these analyses was that naloxone in medical records was administered directly by EMS clinicians; however, we cannot rule out that some portion was administered by bystanders or non-EMS first responders and then subsequently recorded by the EMS clinicians. Any problems in naloxone administration arising from less rigorous medical training for bystanders and non-EMS first responders would bias the results toward no difference between the exposed and unexposed groups. While we were unable to directly assess differential administration and recording practices between EMS agencies, our inclusion of EMS agency as a covariate in our models should help account for regional and agency-level differences in these factors.

While this analysis includes urban and suburban diversity in Northern California, the cohort is limited to a single area of the country, which limits study generalizability. Future studies could address this limitation by including statewide or national datasets.

## Conclusions

In this retrospective cohort of patients with OHCA, EMS-administered naloxone was associated with clinically significant improvements in ROSC and survival to hospital discharge. Additional work is needed to examine the association between naloxone and OHCA outcomes, including prospective interventional studies of naloxone as a potential component of cardiac arrest care.

## Notes

Supplement 1. **eFigure. **Overlap of the Propensity Score Distribution in the Naloxone Exposed and Unexposed Groups, Before and After Matching

Supplement 2.
Data Sharing Statement
