# Supplementary material for: Naloxone and Patient Outcomes in Out-of-Hospital Cardiac Arrests in California
Source: JAMA Netw Open. 2024 Aug 20;7(8):e2429154. doi: 10.1001/jamanetworkopen.2024.29154 (PMC11337064; doi:10.1001/jamanetworkopen.2024.29154)
Supplement: Supplement 1. — eFigure. Overlap of the Propensity Score Distribution in the Naloxone Exposed and Unexposed Groups, Before and After Matching [file jamanetwopen-e2429154-s001.pdf]

Supplemental Online Content

Dillon DG, Montoy JCC, Nishijima DK, et al. Naloxone and patient outcomes in out-of-hospital cardiac arrests in California. *JAMA Netw Open*. 2024;7(8):e2429154. doi:10.1001/jamanetworkopen.2024.29154

eFigure. Overlap of the Propensity Score Distribution in the Naloxone Exposed and Unexposed Groups, Before and After Matching

This supplemental material has been provided by the authors to give readers additional information about their work.

© 2024 Dillon DG et al. *JAMA Network Open*.

eFigure. Overlap of the Propensity Score Distribution in the Naloxone Exposed and Unexposed Groups, Before and After Matching

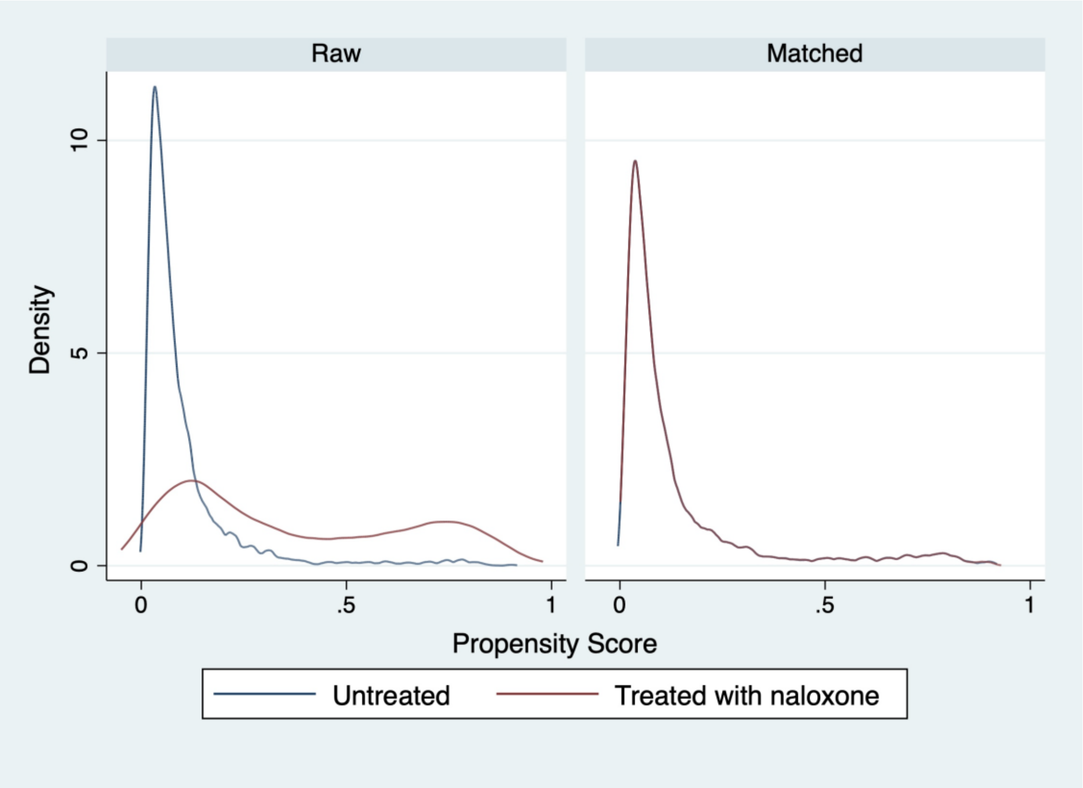

Propensity scores calculated using a regression model with the following variables: age, sex, drug-related cardiac arrest etiology, non-shockable rhythm, co-morbidity, unwitnessed arrest, and emergency medical service agency.

© 2024 Dillon DG et al. *JAMA Network Open*.
