# Supplementary material for: Naloxone and Patient Outcomes in Out-of-Hospital Cardiac Arrests in California
Source: JAMA Netw Open. 2024 Aug 20;7(8):e2429154. doi: 10.1001/jamanetworkopen.2024.29154 (PMC11337064; doi:10.1001/jamanetworkopen.2024.29154)
Supplement: Supplement 2. — Data Sharing Statement [file jamanetwopen-e2429154-s002.pdf]

Data Sharing Statement

Dillon. Naloxone and Patient Outcomes in Out-of-Hospital Cardiac Arrests in California. *JAMA Netw Open*. Published August 20, 2024. doi:10.1001/jamanetworkopen.2024.29154

Data
Data available: Yes
Data types: Deidentified participant data
How to access data: The data that support the findings of this study are available from the corresponding author (djdillon@ucdavis.edu) upon reasonable request.
When available: With publication

Supporting Documents
Document types: None

Additional Information
Who can access the data: Data will be made available to researchers whose proposed use of the data has been approved.
Types of analyses: Data will be made available for approved analyses.
Mechanisms of data availability: Data will be made available with a signed data access agreement after the proposed use of the data has been approved.
